# Pulmonary Vein Isolation Using a Circular Multielectrode Pulsed Field Ablation Catheter via a Jugular Vein Approach

**DOI:** 10.1002/joa3.70239

**Published:** 2025-11-27

**Authors:** Takahiko Nagase, Haruwo Tashiro, Chiyo Yoshino, Ryuichi Kato, Masao Kuwada

**Affiliations:** ^1^ Department of Cardiology Higashiyamato Hospital Tokyo Japan

**Keywords:** atrial fibrillation, interrupted inferior vena cava, pulmonary vein isolation, pulsed field ablation, superior jugular vein approach

## Abstract

PVI by catheter ablation for atrial fibrillation via a superior approach is technically challenging. However, the circular multielectrode PFA catheter is feasible for PVI via a superior jugular vein approach. AP, anteroposterior; LAO, left anterior oblique; PA, posteroanterior; PFA, pulsed field ablation; PVI, pulmonary vein isolation.

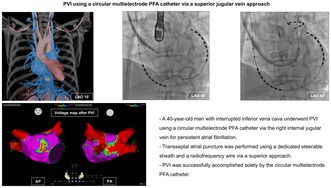

## 1 Introduction

Pulmonary vein isolation (PVI) by catheter ablation for atrial fibrillation via a superior approach in patients with interrupted or congenitally absent inferior vena cava is technically challenging. A previous report described successful PVI using radiofrequency catheters with complex maneuvering [1]. Pulsed field ablation (PFA) has emerged as a major modality for PVI, offering comparable efficacy to thermal energy with reduced collateral injury. A circular multielectrode PFA catheter (PulseSelect; Medtronic, Minneapolis, MN) has shown safety and efficacy in the PULSED AF trial [2]. However, detailed techniques for PVI via a superior approach using this catheter remain unreported. We present a step‐by‐step approach for PVI by the circular multielectrode PFA catheter via a superior jugular vein approach.

## 2 Case Report

**FIGURE 1:**
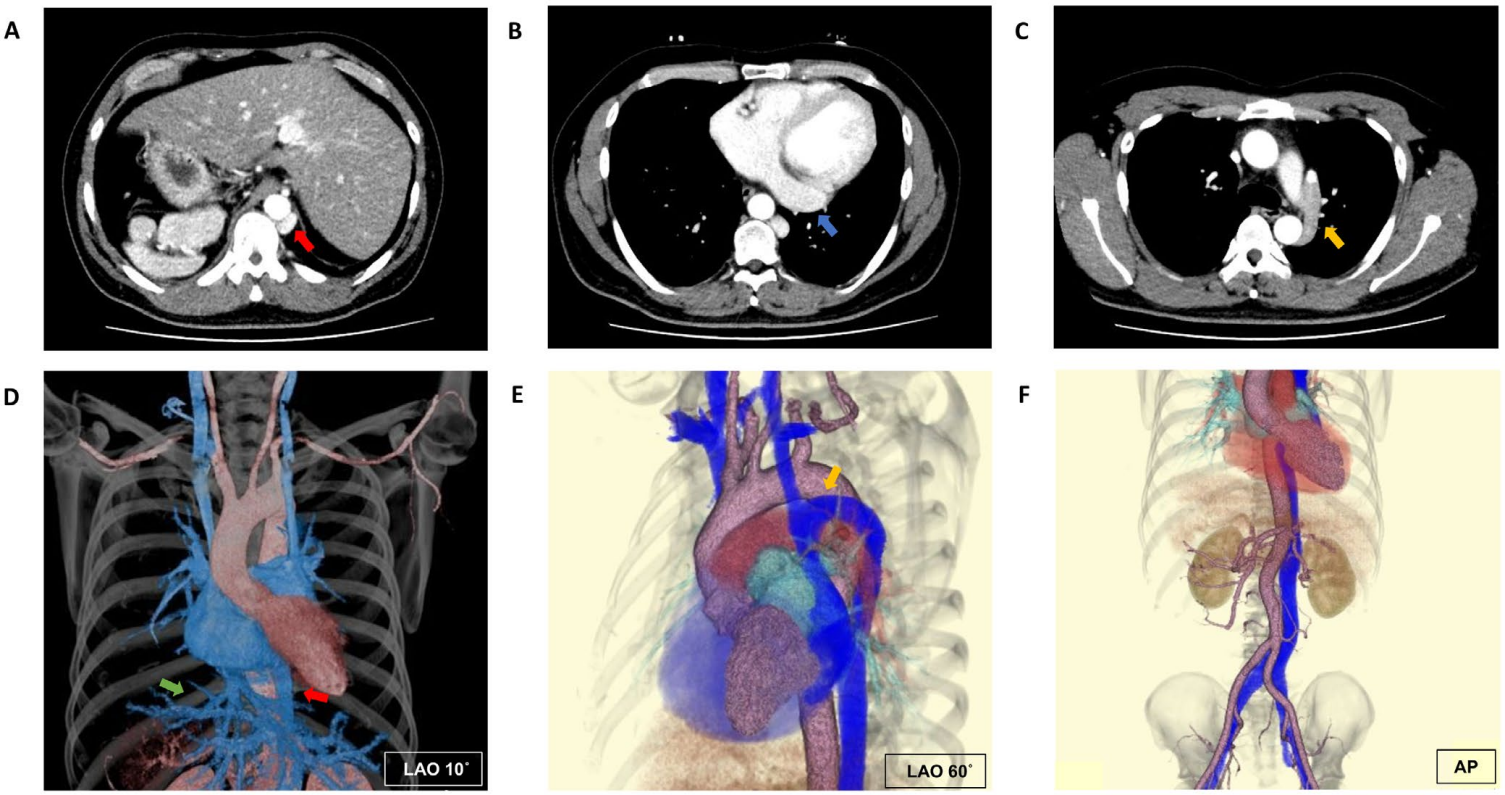
Cardiovascular anatomy. Cross‐sectional views (A–C) and volume‐rendered images (D–F) of the preprocedural computed tomography. Red, blue, yellow, and green arrows indicate interrupted inferior vena cava, persistent left superior vena cava, an azygos continuation to persistent left superior vena cava, and hepatic vein draining into the right atrium, respectively. AP, anteroposterior; LAO, left anterior oblique.

A 40‐year‐old man with mild palpitations, an enlarged left atrial diameter of 41 mm and a mildly reduced left ventricular ejection fraction of 45% underwent catheter ablation for persistent atrial fibrillation. The preprocedural computed tomography revealed an interrupted inferior vena cava with azygos continuation, a persistent left superior vena cava, and partial situs inversus with levocardia (Figure 1A–F).

**FIGURE 2:**
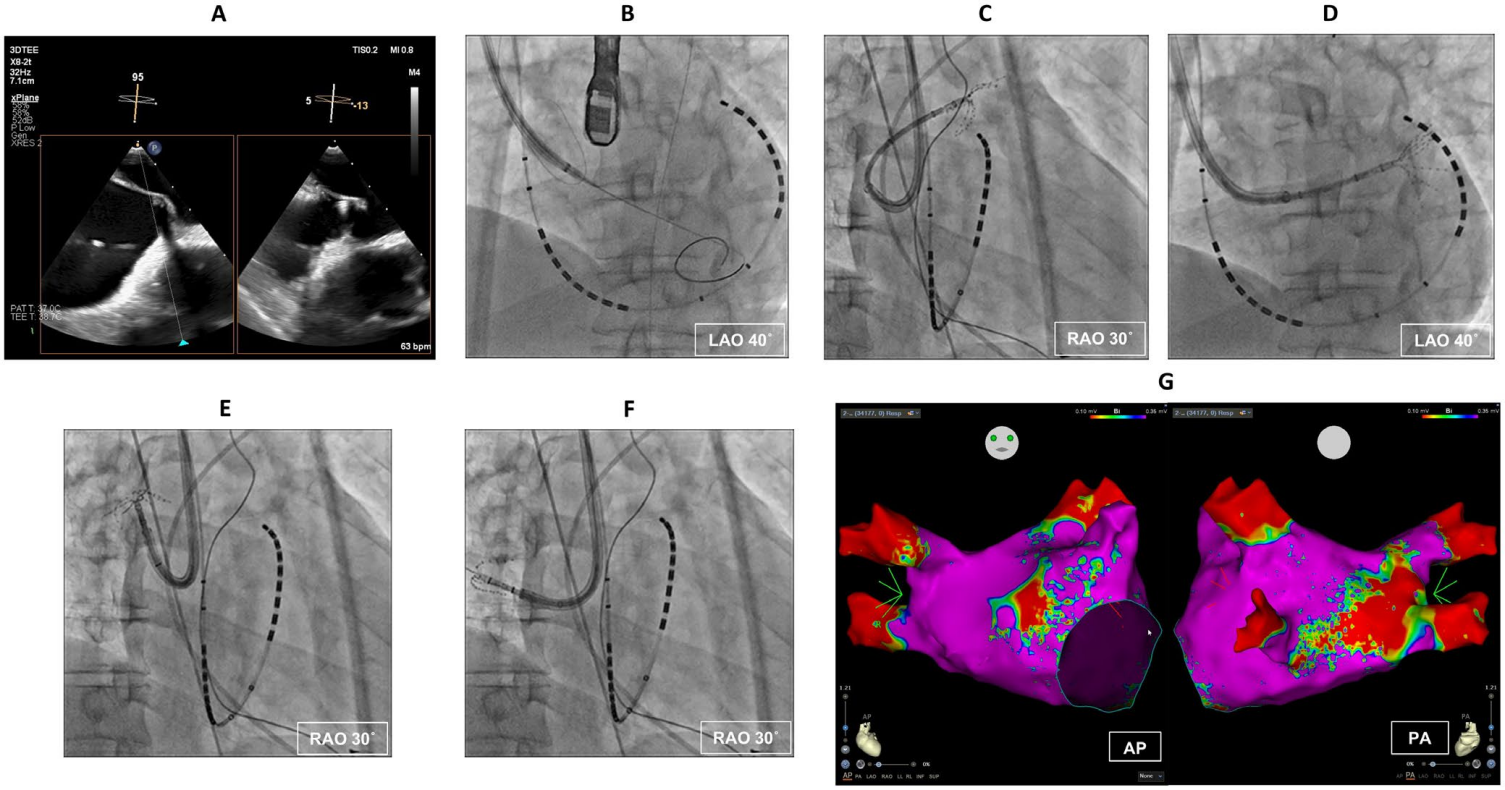
Transseptal puncture and pre‐ablation left atrial voltage mapping. (A) Transesophageal echocardiographic image at the transseptal puncture. (B) Insertion of a radiofrequency wire to left ventricle. (C–F) Voltage mapping of left superior, left inferior, right superior, and right inferior pulmonary veins using a multielectrode mapping catheter (OctaRay, Biosense Webster, Irvine, CA) during atrial fibrillation. (G) Left atrial volage map before ablation. Red and purple colors on the voltage map indicate volages of ≤ 0.1 mV and > 0.35 mV, respectively. PA, posteroanterior; RAO, right anterior oblique; the other abbreviation is defined as in Figure 1.

Transseptal puncture via a superior approach was performed under general anesthesia. A steerable sheath (SupraCross Steerable Sheath, Boston Scientific, Marlborough, MA) was advanced into the right atrium via the right internal jugular vein. A multipolar catheter (BeeAT, Japan Lifeline, Tokyo, Japan) was positioned in the persistent left superior vena cava via the right axillary vein. A 0.025‐inch guidewire was placed at the coronary cusps as an anatomical reference. Transseptal puncture was performed using a radiofrequency wire (SupraCross RF Wire, Boston Scientific), creating tenting of the right atrial septum under transesophageal echocardiography (Figure 2A). The wire was inserted into the left ventricle, following the placement of the sheath into the left atrium (Figure 2B). The steerable sheath was exchanged for a 10F steerable sheath (FlexCath Contour, Medtronic, Minneapolis, MN) via the wire. Before the exchange of the FlexCath Contour sheath, pre‐dilatation of the 8.5F transseptal sheath (SL0, Abbott, Chicago, IL) was required due to resistance at the atrial septum. A high‐density voltage map of the left atrium and all pulmonary veins was acquired using a multielectrode mapping catheter (OctaRay, Biosense Webster, Irvine, CA) integrated with the CARTO3 mapping system (BiosenseWebster) during atrial fibrillation (Figure 2C–F). The left atrial voltage map showed low voltage areas (≤ 0.35 mV) in the left atrial anterior and posterior walls (Figure 2G).

**FIGURE 3:**
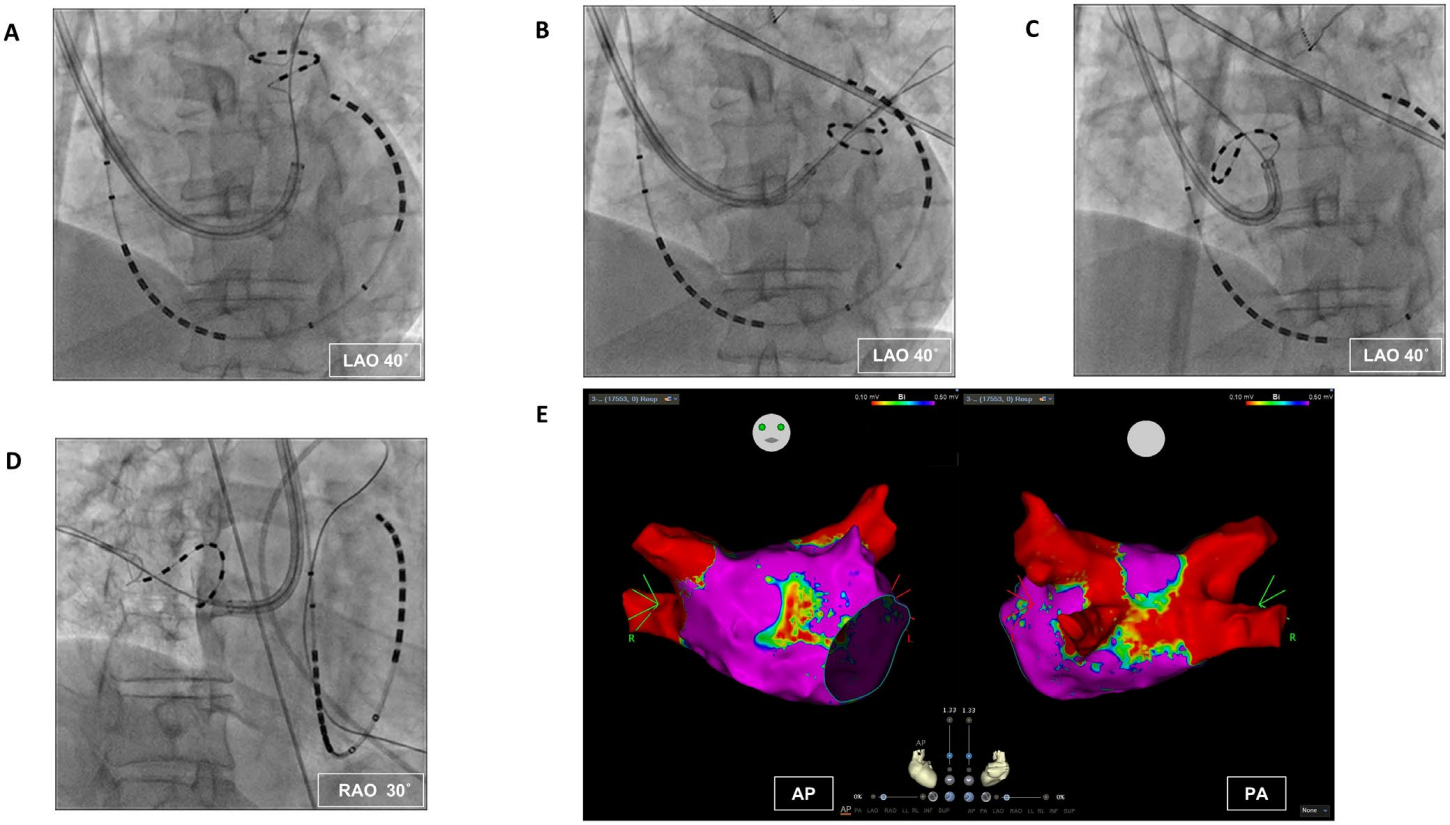
Catheter manipulation using a dedicated steerable sheath. (A–D) Ablation of left superior, left inferior, right superior, and right inferior pulmonary veins. (E) Left atrial voltage map after ablation. Red and purple colors on the voltage map indicate voltages of ≤ 0.1 mV and > 0.5 mV, respectively. Abbreviations are as defined in Figures 1 and 2.

The PulseSelect catheter was advanced via the steerable sheath (FlexCath Contour, Medtronic). In order of left superior, left inferior, right superior, and right inferior pulmonary veins, pulmonary veins were initially ablated by the protocol of 4 ostial and 4 antral applications per pulmonary vein (Figure 3A–D). Additionally, 3 and 5 applications were delivered to the left pulmonary vein carina and the antrum of the right superior pulmonary vein. Atrial fibrillation was converted to sinus rhythm by cardioversion. Left atrial voltage map after PFA using the OctaRay catheter during sinus rhythm showed complete antral PVI without additional radiofrequency ablation (Figure 3E). While the final voltage map showed some heterogeneity along the inferior line of the left atrium, the procedure was concluded without additional ablation due to no inducibility of atrial tachyarrhythmia after PVI, the patient's young age, and prolonged procedure time.

Due to anatomical complexity, the time from jugular venous access to insertion of the FlexCath Contour sheath was prolonged to 194 min. This delay was primarily attributable to (1) difficulty in visualizing the atrial septum, which was obscured by the esophagus coursing along a mid‐to‐right trajectory behind the left atrium in the setting of partial situs inversus with levocardia, and (2) multiple adjustments of the puncture site and the need for pre‐dilatation with an 8.5F transseptal sheath due to a thick and resistant atrial septum. Consequently, the total procedure time and the time from jugular access to completion of PVI were 313 and 246 min, respectively. Notably, once the FlexCath Contour sheath was successfully positioned, left atrial voltage mapping and PVI were completed in 16 and 52 min, respectively. The patient was discharged without any complication.

## 3 Discussion

The PulseSelect catheter is a 9F over‐the‐wire catheter with a 9‐electrode circular design, 25‐mm diameter, electrode spacing of 3.75 mm, and electrode width of 3 mm. The size of the catheter and the 10F dedicated sheath (FlexCath Contour, Medtronic) was suitable for internal jugular vein approach for minimizing access site injury, whereas another PFA system requires a thicker dedicated sheath (e.g., 13F steerable sheath, FARADRIVE, Boston Scientific, Menlo Park, CA) [3]. Additionally, a thinner sheath may be preferred, because the insertion of the sheath through the atrial septum may require larger tip load via a superior approach than a normal approach from femoral veins. The over‐the‐wire catheter system also facilitated both precise navigation toward the pulmonary veins and smooth rotational control at the ostia and antra compared with another PFA catheter (e.g., VARIPULSE, Biosense Webster) and conventional radiofrequency [4]. These advantages were particularly beneficial in the context of complex anatomy and superior venous access. Cryoballoon ablation via the jugular approach has also been successfully reported and may offer procedural efficiency [5]. In this case, however, cryoballoon was not selected due to concerns regarding sheath size (12 Fr, FlexCath Contour, Medtronic) and the need for stable coaxial positioning, which can be technically challenging via the superior route. In addition, compared to the previous report of manual radiofrequency ablation via a superior approach, where durations from transseptal puncture to the end of the procedures ranged widely from 104 to 475 min due to complex catheter manipulation [1], the use of PFA with a circular multielectrode catheter may facilitate a more streamlined workflow and relatively shorter ablation time.

## 4 Conclusion

This case demonstrates the feasibility and safety of PVI via a superior jugular vein approach using a circular multielectrode PFA catheter. The techniques offer a viable alternative in patients with interrupted inferior vena cava, with favorable catheter maneuverability and procedural efficiency.

## Funding

The authors have nothing to report.

## Ethics Statement

This study was conducted in accordance with the ethical principles of the Declaration of Helsinki.

## Consent

Written informed consent was obtained from the patient for the publication of this case report.

## Conflicts of Interest

The authors declare no conflicts of interest.

## Data Availability

The data that support the findings of this study are available from the corresponding author upon reasonable request.
